# COVID-19 in comparison with other emerging viral diseases: risk of geographic spread via travel

**DOI:** 10.1186/s40794-020-00129-9

**Published:** 2021-01-31

**Authors:** A. Wilder-Smith

**Affiliations:** 1 Department of Disease Control, London School of Hygiene and Tropical Medicine, London, UK; 2 Heidelberg Institute of Global Health, University of Heidelberg, Heidelberg, Germany

**Keywords:** SARS, Measles, Dengue, Zika, Chikungunya, Yellow fever, West Nile encephalitis, Japanese encephalitis, Ebola, Monkeypox

## Abstract

**Purpose of review:**

The COVID-19 pandemic poses a major global health threat. The rapid spread was facilitated by air travel although rigorous travel bans and lockdowns were able to slow down the spread. How does COVID-19 compare with other emerging viral diseases of the past two decades?

**Recent findings:**

Viral outbreaks differ in many ways, such as the individuals most at risk e.g. pregnant women for Zika and the elderly for COVID-19, their vectors of transmission, their fatality rate, and their transmissibility often measured as basic reproduction number. The risk of geographic spread via air travel differs significantly between emerging infectious diseases.

**Summary:**

COVID-19 is not associated with the highest case fatality rate compared with other emerging viral diseases such as SARS and Ebola, but the combination of a high reproduction number, superspreading events and a globally immunologically naïve population has led to the highest global number of deaths in the past 20 decade compared to any other pandemic.

## Key points

1. Public health emergencies of international concern in the past 20 years include COVID-19, poliomyelitis, H1N1, Ebola and Zika
2. COVID-19 is the worst pandemic in scale and speed of this century associated with the highest number of global deaths
3. Spread via air travel is most striking for respiratory pathogens rather than vector-borne or other emerging viruses
4. Combating COVID-19 will require an all-society and all-government approach

## Introduction

Emerging infectious disease outbreaks are most likely to originate in wildlife, and are increasing significantly over time correlated with socio-economic, environmental, ecological factors combined with increasing mobility and globalization [1, 2] including climate change [3, 4]. Viral outbreaks differ in many ways, such as the individuals most at risk e.g. pregnant women for Zika and the elderly for COVID-19, their vectors of transmission, their fatality rate, and their transmissibility often measured as basic reproduction number. Despite these differences, policy responses used to tackle viral epidemics have tended to be similar across time and countries – social distancing, quarantines, school closures [5], and information campaigns are the policy instruments normally available, in addition to vector control campaigns for vector-borne viral diseases, personal protection, and most importantly, vaccines. For diseases with high potential of geographic spread associated with high case fatalities, rigorous mobility and travel restrictions are being deployed [6]. This review examines emerging or re-emerging viral infections of the past two decades, their characteristics and the risk of geographic spread via air travel.

## Coronaviruses

The current pandemic is caused by a coronavirus of zoonotic origin -SARS-CoV-2-, that emerged in Wuhan, China, by the end of 2019, and was rapidly declared a public health emergency of international concern (PHEIC). SARS-CoV-2 is most likely of bat origin, similar to its predecessor SARS virus that caused the SARS outbreak in 2003 [7]. Live animal markets selling multiple species of wild and domestic animals in proximity to large populations of densely housed humans are thought to be the source of both outbreaks [8]. The main transmission route is via respiratory droplets, and the angiotensin converting enzyme 2 (ACE2), found in the lower respiratory tract of humans, has been identified as the receptor used for cell entry for both SARS and SARS-CoV-2 [9, 10]. The basic reproductive number R0 is 2–3, indicating that every case leads to 2–3 secondary cases), is similar or somewhat higher to that of SARS [11]. R0 above 1 will lead to propagation and further growth of the outbreak.

Risk factors for severe disease outcomes include older age and co-morbidities. The higher asymptomatic rate, further compounded by pre-symptomatic transmission has made containment much harder for COVID-19 than for SARS [8]. COVID-19 spread is facilitated by population densities, urbanization, mass gatherings and superspreading events [12–15]..

The SARS epidemic in 2003 reported 8098 cases with 774 deaths, and was eventually brought under control by July 2003, in a matter of 8 months [8]. By interrupting all human-to-human transmission through aggressive case detection, prompt isolation, contact tracing with legal enforcement of quarantine, SARS was effectively eradicated [8]. In contrast, by December 2020, SARS-CoV-2 has caused more than 70 million infections with more than 1.7 million deaths, in a matter of 12 months with no sign of abating. Although lockdowns and unprecedented travel restrictions were able to flatten the curve, pre-mature re-opening led to resurgence in most countries [16].

The Middle East Respiratory Syndrome Coronavirus (MERS-CoV) has plagued the Middle East since it was first reported in 2012. MERS spread to 27 countries across the globe, with more than 2274 cases with 842 case fatalities to date [17]. Similar to COVID-19, household transmission, transmission to health care workers and clustering are common [18]. Cross-species transmission is the likely origin of this virus. Camels may act as a direct source of human MERS-CoV infection [19]. The case fatality rate of MERS (37%) is far higher than that of COVID-19, yet, outbreaks were contained relatively expediently as they were usually limited to hospital-based outbreaks.

### Geographic spread of coronaviruses via air travel

Prior to the lockdown in Wuhan, China, COVID-19 rapidly spread within China, and also globally following the direction of connectivity and high air passenger volumes [20, 21]. The epicenter from Wuhan quickly moved to Iran, then Italy, and then all of Europe, followed by increasingly bigger outbreaks in the United States, Brazil and other countries in the Americas. Mass gathering events and spread via returning travelers or pilgrims triggered new outbreaks in various countries [22–24]. The vast majority of countries in the world now report COVID-19 cases, although some countries have been more successful than others in implementing public health measures to curb the epidemic, such as China, Taiwan, Vietnam, Thailand, New Zealand and Singapore [25]. Travel bans have led to a delay of importation into Australia [26]. Entry and exit screening, quarantine for 14 days, and increasingly testing is being used to identify imported cases and prevent onward transmission [27].

Although SARS spread to 26 countries, the vast majority of cases were concentrated in five countries or regions: China, Taiwan, Hong Kong, Singapore and Toronto, Canada. Cluster effect and superspreading events were well described, but community transmission only occurred to a larger extent in China—all other countries had minimal community transmission, and the majority of transmission occurred within hospitals. Through rigorous public health measures such as prompt institutional isolation of all cases, contact tracing and quarantine (with legal monitoring) for 14 days of all contacts, and personal protective measures in hospitals, SARS was effectively eradicated. Although travel warnings were issued, no lockdowns were implemented except in China. Most of the outbreaks were nosocomial.

With regards to MERS, Saudi Arabia (KSA) so far has carried the greatest brunt of MERS-CoV since its emergence, with 85% of the total global reported cases being either diagnosed or originating in KSA, with a total of 1897 cases and 734 fatalities. The second major outbreak was due to exportation of MERS from Saudi Arabia via a business traveler to South Korea resulting in an explosive but in the end contained outbreak in 2015 [28]. Lessons learned from the MERS outbreak were the foundation of Korea’s enhanced pandemic preparedness plans which enabled the country to successfully curb the COVID-19 outbreak in Korea in early 2020.

## Ebola

Ebola was declared a public health emergency in 2014 in West Africa, and again in 2018 in the Democratic Republic of Congo [6, 29]. In the pantheon of emerging infectious diseases, Ebola virus stands out as one of the deadliest. The Zaire species of Ebola kills somewhere between 40 to 90% of its victims, and usually upwards of 60% of infected people die [30]. Only a handful of infectious diseases can claim such high death rates, including rabies [31, 32], pneumonic plague, and inhalational anthrax [33]. About 14,000 deaths due to Ebola were recorded in 2014. Why did the Ebola outbreak result in a much lower death toll compared to COVID-19? One major difference between Ebola and COVID-19 is the mode and timing of transmission. Ebola is spread during the last stage of the disease through bodily fluids. Exposure of infected individuals to a high-density population could result in a catastrophic outbreak, however, overall the R0 is far lower than that of COVID-19 as transmission depends on closer proximity between humans, in particular contact to bodily fluids. Those persons who are at greatest risk for Ebola infection are those who have very close contact taking care of the sick, bedridden victims, regardless whether they are in the home or the hospital. Rapid patient identification, isolation and aggressive follow up is shown to rapidly limit the potential for disease spread. Isolation of Ebola cases is successful because of the absence of pre-symptomatic shedding, eg the virus only or mainly spreads when the victim has symptoms. Transmission can also occur because of reservoirs of the virus in survivors in their eyes or semen.

The world’s second largest Ebola outbreak occurred in the Democratic Republic of Congo (DRC) in 2018 and led to a second declaration of a public health emergency in 2019. The public health emergency was declared over on 25 June 2020. The nearly two year-long outbreak was particularly challenging because it took place in an active conflict zone. Led by the Government and the Ministry of Health of DRC and supported by WHO and partners, the response involved training thousands of health workers, registering 250,000 contacts, testing 220,000 samples, providing patients with equitable access to advanced therapeutics, vaccinating over 303,000 people with the highly effective Ebola vaccine (rVSV-ZEBOV-GP vaccine) [34, 35], and offering care for all survivors after their recovery. The response was bolstered by the engagement and leadership of the affected communities [36]. By July 2020, a total of 3481 cases (3323 confirmed, 158 probable) with 2299 deaths had been reported [36].

### Geographic spread of Ebola via air travel

Spread of Ebola occurred mainly via land border crossings between West African countries in 2014. Spread via air travel has only rarely occurred, the main reason being that Ebola patients are so sick that they cannot board a plane or are picked up at entry screening. However, since Ebola has an incubation period of up to 21 days, carriers could arrive in a country weeks before symptoms develop – potentially transmitting it to the people they know. Eleven Ebola cases were reported in the US in 2014, of which seven cases were medically evacuated from other countries. Nine of the people contracted the disease outside the US and traveled into the country, either as regular airline passengers or as medical evacuees; of those nine, two died. Two people contracted Ebola in the United States. Both were nurses who treated an Ebola patient; both recovered. In 2014 some US state governors signed an order authorizing the mandatory quarantine for 21 days of anyone, even if asymptomatic, who had direct contact with Ebola patients, over and beyond the CDC’s voluntary quarantine.

Based on epidemic conditions and international flight restrictions to and from Guinea, Liberia, and Sierra Leone as of Sept 2014, models projected 2.8 travellers infected with Ebola virus departing the above three countries via commercial flights, on average, every month. 91,547 (64%) of all air travellers departing Guinea, Liberia, and Sierra Leone had expected destinations in low-income and lower-middle-income countries [37]. Screening international travellers departing three airports would enable health assessments of all travellers at highest risk of exposure to Ebola virus infection. For the Kivu outbreak in DRC in 2018, studies showed little commercial airline connectivity from the Ebola-affected area; however, larger cities in DRC and throughout East Africa should be aware of the potential for Ebola virus importation through this route [38]. Due to limited air travel from the DRC, the outbreak did not spread globally [38].

## H1N1 influenza

Influenza attack rates vary by season, by geographic location, by setting (eg closed settings versus community settings), by predominant subtype and by age group. Influenza outbreaks have been described in hospitals, aboard cruise ships [39] and on airplanes [40]. H1N1 preceded the PHEIC declaration of Zika, and was the cause of the 2009 pandemic. The 2009 H1N1 pandemic strain possessed a unique combination of gene segments including genes that originated from swine, avian and human influenza viruses that had been circulating among pigs in North America and Europe [41]. The age and mortality risks for the H1N1 influenza pandemic were different to the current COVID-19 pandemic, with younger persons affected but an overall much lower case fatality rate than COVID-19. The epidemic was focused in children, with an effective reproduction number of approximately 1.2–1.3 [30] compared to 2.5 to 3.2 for SARS-CoV-2 [11]. During 2009, the first year after the emergence of the virus, an estimated 62 million illnesses, 274,000 hospitalizations, and 12,400 deaths associated with the 2009 H1N1 virus occurred in the United States [30]. The highest attack rates were in children younger than 5 years of age, and 90% of estimated deaths occurred in persons younger than 65 years of age. In one estimate, the mean age among persons whose deaths were attributed to H1N1 was 37 years, compared to a mean age of 76 years among deaths attributed to seasonal influenza. While vaccine was delivered relatively late, it was demonstrated to be effective. Excess deaths attributed to influenza varied from 3300 deaths to 48,000 deaths [30]. FluNet is the main tool for information sharing among the WHO Global Influenza Surveillance Network, as well as the public. It allows 112 WHO National Influenza Centers in 83 countries access to remote data entry [42].

### Geographic spread of influenza, in particular H1N1, via air travel

The role of air travel in the global spread of influenza has been the subject of a significant body of research. H1N1 spread rapidly according to air travel patterns first from Mexico to the United States and then around the globe during the initial wavefront of this epidemic [43]. The speed and direction of spread is directly correlated with air passengers volumes and destinations of the majority of flights. Given the short incubation time, isolation and quarantine is much less effective compared to diseases with a longer incubation time such as SARS and COVID-19. Regarding air travel, the main route connected to the influenza source area should be targeted for travel restrictions. Imposing a 99% air travel restriction delays the epidemic peak by up to 2 weeks [44]. Simulation modelling for the 1998–1999 through 2000–2001 influenza seasons using a standard compartmental model coupled with air transportation data showed that air travel plays an important role in the spread of annual influenza within the U.S., particularly in cities with large air travel volumes [45]. Combination strategies can delay spread, reduce overall number of cases, and delay and reduce peak attack rate more than individual strategies [46]. However, antivirals and hospitalization were found to be more effective on attack rate reductions than travel restrictions. Combined strategies (with 99% restriction on all transport modes) deferred the peak for long enough to establish a vaccination program [44].

A stochastic discrete event simulation model assessed the effectiveness of passenger screening at US airports [47]. Modelling of US airport screening would identify 50% infected individuals; efficacy is limited because of asymptomatically infected passengers. Screening would not significantly delay arrival of pandemic influenza via international air transport but could reduce the rate of new US cases and subsequent deaths. Implementation of entry screening policies was associated with on average additional 7–12 day delays in local transmission compared to nations that did not implement entry screening, with lower bounds of 95% confidence intervals consistent with no additional delays and upper bounds extending to 20–30 day additional delays [48]. The resources required for implementation should be balanced against the expected benefits of entry screening. A study in China during May–November 2009 analysing the effectiveness of border entry screening and holiday-related school closures, age distribution and transmission dynamic characteristics were similar to those in Northern Hemisphere temperate countries. The 8 days of national holidays in October reduced the effective reproduction number by 37% (95% credible interval 28–45%) [49]. Restrictions on air travel are projected to be of “surprisingly little value in delaying epidemics, unless almost all travel ceases very soon after epidemics are detected.” Interventions to reduce local transmission of influenza are likely to be more effective at reducing the rate of global spread [50].

## Measles

Measles is one of the most transmissible viral infections that although mild in most cases, can cause serious illness, life-long complications and death [51]. Measles is transmitted from human to human via respiratory droplets, and is associated with the world’s highest reproduction number of more than 10 [30], far higher than that of SARS-CoV-2. Strebel in “Plotkin’s Vaccines” reports that the case fatality ratio is high in children aged < 1 year, lower in children aged 1–9 years, and then rises again in teenagers and adults [30]. In industrialized countries, death occurs in 1–3 out of 1000 cases. Measles runs a more devastating course in children in developing countries or in settings with minimal care where measles mortality rates can be as high as 2 to 15% [30]. Before vaccine introduction, measles affected over 90% of children globally by the age of 15 [52]. Effectiveness of the measles vaccine after two doses is as high as 97% [53]^.^ Given such an efficacious vaccine, in 2012, the World Health Assembly set the objective of eliminating measles in four of the six World Health Organization (WHO) regions by 2015 and in five regions by 2020 [54]. Countries in all six WHO regions have adopted goals for measles elimination by 2020. Indeed, during 2000–2017 annual reported measles incidence decreased 83% because of vaccination campaigns and childhood vaccination programmes, from 145 to 25 cases per million population; and annual estimated measles deaths decreased 80%, from 545,174 to 109,638 [52].

The declaration of measles elimination from the Americas in 1999 was a historic milestone, as the first WHO region to eliminate measles [55]. However, in 2018, the Americas saw a major resurgence, mainly because of the migration crisis in Venezuela [56, 57], combined with decreasing vaccine coverage that increased the vulnerability to importation of measles [58]. The extent of the current outbreak is a setback to the WHO’s global measles elimination goals. In 1998, the European Region WHO set a target to eliminate measles and rubella by 2010 [59], which was not achieved. In 2018, Europe saw a major resurgence of measles; the total number of measles cases in 2018 was the highest in this decade, reaching three times the total cases in 2017 [60]. In 2018, Europe reported more than 21,000 cases of measles, including 35 deaths [61]. Early reports in 2019 show a further 300% increase [62]. Of note, social distancing and lockdown measures during 2020 resulted in a major decline of measles [63].

As there is no animal reservoir for measles, measles resurgence is due to human movement of viraemic persons [61]. Some attribute the enormous migration into Europe in the past 5 years fas the reason for the measles resurgence in this region [64–66]. Indeed, infectious diseases in migrants including measles are well documented [55, 67–71]. Travelers and pilgrims also play a role in disease propagation, including adoption from measles-endemic countries [72–74]: However, the main reason for the resurgence of measles globally is vaccine hesitancy leading to suboptimal vaccine coverage rates [75]. In the year 2020, measles vaccine coverage rates dropped further due to the COVID-19 pandemic as lock-downs interrupted routine immunization programmes and a further resurgence of measles is expected in the years to come [76]. The colliding epidemics of COVID-19, measles and Ebola in DRC are of particular concern [77].

### Geographic spread of measles via travel

Measles is often not considered a risk for travellers, and hence pre-travel advice often does not include measles vaccination [71, 78]. In a study among returned Australian travellers, only 1 of 25 imported cases reported seeking pre-travel health advice and few had perceived measles as a travel-associated disease [79]. Measles was also highlighted as a high risk for amplification during the Hajj pilgrimage [74, 80]. While there are reports of measles importations resulting from international adoptions and humanitarian entrants, the majority of international travel and subsequently, the majority of importations of measles are in short-term travellers [81]. Large, sustained outbreaks in countries with sub-optimal immunisation coverage, such as many countries in Europe, result in regular incursions by travellers into regions that have eliminated measles, some resulting in local outbreaks.

Migrants are often unfairly blamed for the spread of measles. However, the estimated seroprevalence of measles IgG antibodies of 80–88% among recent migrants from the WHO African region [66], while sub-optimal, these rates are greater than WHO coverage estimates from the region and on par with coverage reported in some European receiving countries. Despite record numbers of migrants arriving in Europe, the contribution of migration on the current epidemiology of measles in Europe is minimal [55]. However, immigrants from within and outside Europe are a growing population group and face barriers to accessing immunisation and other health services and strategies to reach migrant populations and provide catch-up immunisation are needed [55].

## Poliomyelitis

Since the Global Polio Eradication Initiative was established in 1988, two of the three wild poliovirus (WPV) serotypes (types 2 and 3) have been eradicated. Transmission of WPV type 1 (WPV1) remains uninterrupted only in Afghanistan and Pakistan. Although the case fatality rate is very low, the resulting life-long disabilities. Poliomyelitis was the leading cause of permanent disability in children in the pre-vaccine era. In 1988, when the global eradication target was adopted, approximately 350,000 cases of paralytic poliomyelitis were occurring annually. In 2019, Afghanistan and Pakistan reported the highest number of WPV1 cases (176) since 2014. During January 1–March 31, 2020 (as of June 19), 54 WPV1 cases were reported, an approximate fourfold increase from 12 cases during the corresponding period in 2019 [82]. Paralytic poliomyelitis can also be caused by circulating vaccine-derived poliovirus (cVDPV), which emerges when attenuated oral poliovirus vaccine (OPV) virus reverts to neurovirulence following prolonged circulation in under-immunized populations. Since the global withdrawal of type 2-containing OPV (OPV2) in April 2016, cVDPV type 2 (cVDPV2) outbreaks have increased in number and geographic extent. During January 2018–March 2020, 21 countries reported 547 cVDPV2 cases [82]. The COVID-19) pandemic and lockdowns have resulted in suspension of immunization activities and disruptions to poliovirus surveillance in 2020.

### Geographic spread of polio via travel

Poliomyelitis is a rare disease on a global scale, and hence spread via travel is rare. That said, the endgame of polio eradication is hampered by the international spread of poliovirus via travelers [83]. In response to ongoing importations of poliovirus into polio-free countries, on 5 May 2014, WHO declared the international spread of wild poliovirus a public health emergency of international concern. Unbeknown to many, polio is still a public health emergency of international concern.

In a modelling study on the risk of exportation of poliomyelitis, immunization coverage rates, infectious period, the asymptomatic-to-symptomatic ratio, and also the probability of a traveler being infectious at the time of travel were considered [83]. The model estimated 665 polio exportations (> 99% of which were asymptomatic) from nine polio-infected countries in 2014, of which 78.3% originated from Pakistan [83]. This model also estimated 21 importations of poliovirus into Saudi Arabia via Hajj pilgrims and 20 poliovirus infections imported to India in the same year. For countries that are vulnerable to polio outbreaks due to poor national polio immunization coverage rates, this model may help guide policy makers to decide whether imposing an entry requirement in terms of proof of vaccination against polio would be justified, as India did for Pakistan when India was declared polio-free.

## Monkeypox and smallpox

The identification of monkeypox imported in two separate travelers to the United Kingdom with one onward transmission to a health care worker in 2019 raised media and political attention on an emerging public health threat [84]. In the same year, Nigeria experienced an unusual outbreak of monkeypox, after the case was confirmed in 1978. As of 13th October 2018, there have been one hundred and sixteen confirmed cases the majority of whom are under the age of 40 years [84]. First identified in the Democratic Republic of Congo (DRC) in 1970, monkeypox has since 2010 expanded to cause outbreaks among humans in at least seven additional African countries: Cameroon, Central African Republic, Republic of the Congo, Liberia, Nigeria, Sierra Leone and South Sudan [85] Major knowledge gaps remain on the epidemiology, host reservoir, and emergence, transmission, pathogenesis and prevention of monkeypox. Vaccine development against monkeypox is ongoing [86]. While monkeypox is currently not a global threat, many lessons can be learned from its “cousin” virus smallpox on the need for international cooperation and a well-funded global vaccination programme was needed to eradicate a disease [87]. It is estimated that over 70% of the world’s population is no longer protected against smallpox, and through cross-immunity, and therefore also not to monkeypox.

### Geographic spread of monkeypox via travel

The importations to the UK and an importation to Israel represent the first-time international travellers have been implicated in the spread of monkeypox outside of an outbreak setting [50]. In 2003, US several human monkeypox cases traced to virus exposure via infected captive prairie dogs were reported [88]. The virus was likely introduced through a shipment of imported African rodents, which were kept with other mammals, including prairie dogs, in a pet distribution facility in the Midwest. To prevent further monkeypox virus introduction, importation of African rodents was restricted and restrictions were introduced on domestic trade or movement of prairie dogs and certain other rodents.

## Emerging arboviruses

Arthropod-borne viruses (arboviruses) have a long history of emerging to infect humans, but during recent decades, they have been spreading more effectively with widening of the geographic distribution, and increasing magnitude and frequency of outbreaks [89]. This is due to several factors, including increased air travel, climate change [90] and population growth including urbanization [91]. Urbanization is particularly important for the re-emergence of dengue, whereby humans living in close proximity become the amplification hosts and peri-domestic mosquitoes, mainly *Aedes aegypti*, mediate human-to-human transmission. Dengue, yellow fever, chikungunya, and Zika viruses have undergone such urban emergence. Emergence can involve simple spill-over from enzootic (wildlife) cycles, as in the case of West Nile virus accompanying geographic expansion into the Americas, and recently also increasingly in Europe [92]; secondary amplification in domesticated animals, as seen with Japanese encephalitis [93], Venezuelan equine encephalitis, and Rift Valley fever viruses [94].

The Zika outbreak in the Americas was declared a PHEIC in January 2016. The Zika epidemic presents the first ever known association between a flavivirus, carried by the *Aedes aegypti* mosquito, and congenital disease. The congenital disease most closely linked to the Zika virus in the 2015 epidemic was microcephaly where the occipitofrontal head circumference is smaller than 98% of all newborns, but many more neurodevelopmental anomalies were observed [95, 96]. Mortality in adults is extremely low. Zika was also unusual as for the first time in history sexual transmission of a flavivirus was confirmed. Sexual transmission also increased the fear of importation and subsequent forward transmission even where the vector does not exist, and prompted WHO and CDC to publish advice on how to reduce the risk of sexual transmission in travelers [97]. Although Zika is best documented for its associated with maternal infections and their impact on birth defects, also dengue and chikungunya can lead to maternal complications as well as severe infections in the neonate [98].

Dengue is the most prevalent arboviral disease, present in more than 120 countries affecting more than 2 billion people [99]. The most common life-threatening clinical response to dengue infection is the dengue vascular permeability syndrome, epidemiologically linked to secondary infection, but can also occur in primary infection. Antibody-dependent enhancement, viral factors, age, host factors, and clinical experience of the managing physician modulate the risk of progressing to severe dengue. The reported relative risk of severe dengue in secondary versus tertiary infection ranges from 2 to 7 [100]. The absolute risk of severe dengue in highly endemic areas in children is about 0.1% per year for primary infections, and 0.4% for secondary infections. About 2–4% of secondary infections lead to severe dengue. Clinical management of severe dengue depends on judicious use of fluid rehydration. The risk of travel-acquired dengue depends on destination, season and duration of travel and activities during travel. Seroconversion rates reported in travellers therefore vary between less than 1% to more than 20% [100]. Chikungunya is associated with a low mortality, but high morbidity with disabling arthritis that can last for months beyond the viremic phase [101]. On the other hand, yellow fever has the highest case fatality rate and is therefore one of the most feared arboviral diseases [102].

### Geographic spread of arboviral diseases via travel

The spread of arboviral diseases via travelers to non-endemic countries is well documented, and has led to the geographic expansion including new establishment in countries where the vector exists. The rapid geographic spread of dengue viruses globally is the result of increasing mobility of people via modern means of transportation [103–106]. Air travel connectivity between dengue-endemic countries and from dengue-endemic countries to non-endemic, but still vulnerable settings has increased exponentially [107]. Whilst imported dengue cases to the US have resulted in small dengue clusters for many years [108]; the first autochthonous sporadic cases in Europe (France and Croatia) were reported only in 2010 [109, 110]. In 2012, the first major European outbreak of dengue occurred in Madeira [111]. Viremic travelers to non-endemic areas where *Aedes* mosquitoes exist constitute the source for triggering autochthonous transmission [112]. About 58% of travelers who returned to Europe after acquiring a dengue infection during their travel to dengue-endemic countries were viremic when seeking medical care, thus highlighting the potential for dengue virus introduction [113]. Fortunately, the seasonal window in Europe when vectorial capacity is sufficient to sustain autochthonous transmission is short [114].

A statistically significant positive association between passenger flows via airline travel from countries experiencing chikungunya epidemics and the number of imported cases in the USA at the state level [115]. This validation study demonstrated that air travel was strongly associated with observed importation of chikungunya cases in the USA and can be a useful proxy for identifying areas at increased risk for disease importation. For the first time in history, in 2016, yellow fever was exported from Africa to Asia [116].

Spread of arboviral diseases via air travel is mainly driven by viremic travelers, but spread can also occur through infected mosquitoes. Insecticide treatments in aircraft (termed ‘aircraft disinsection’) aim to support the containment of potentially disease-carrying vector insects. Despite established efficacy of aircraft disinsection in trials, its effectiveness and feasibility in flight operations, and its usefulness as a public health measure need to be enhanced [117].

### Stakeholders of pandemics

Numerous travel stakeholders are affected by, and affect, pandemics. Although the key purpose of the International Health Regulations (IHR) is to prevent unwarranted interruptions to trade and travel during large transnational infectious disease outbreaks [29], stakeholders react in different ways depending on political pressure, public sentiment and the media. For example, WHO did not issue any travel restrictions for the Ebola outbreak, yet air travel plummeted. The reasons for interruption of travel during the 2014–16 Ebola outbreak were complex, with decisions by States only partly contributing to the cessation. Decisions by non-state actors, particularly the travel industry itself, were major drivers [6].

### Risk of in-flight transmission

More than 1 billion passengers travel by air annually [107]. Although this manuscript focuses on the risk of spread via air travel, it is important to also quickly mention transmission that may happen on flights. In-flight transmission can occur especially for respiratory pathogens: Airborne transmission may occur through large droplets > 5 μm that fall to the floor within a radius of 1 m and might be better called contact transmission, or through aerosolization of an infectious agent in droplets < 5 μm that remain airborne for a long time and can disperse widely [40]. Cabin air is normally exchanged 15 to 20 times per hour, as compared with 12 times per hour in most office buildings. Aircraft recirculates 50% of the air to control humidity and fuel efficiency. Recirculated air passes through high efficiency particulate air (HEPA) filters capable of removing 99.97% of particles 0.3 μm in diameter, hence it is not the air quality but the proximity of persons that present the greatest risk of in-flight transmission. Precise data on airborne transmission of infections in aircraft are scarce and imprecise, but they suggest that risk is associated with sitting within 2 rows of a contagious passenger for a flight time of more than 8 h [40]. This association is mainly derived from studies of in-flight transmission of *Mycobacterium tuberculosis* in which tuberculin skin test conversion was taken to indicate infection acquired during flight. In one study, 4 of 15 passengers within 2 rows had a tuberculin skin test conversion [40]. During the SARS outbreak in 2003, 40 flights were identified with a case of SARS on board; in-flight transmission is thought to have taken place on 5 of these flights, infecting a total of 37 passengers [40]. As for COVID-19, in-flight transmission has been reported for more than 40 flights, but the number could be much higher as ascertainment to in-flight transmission is difficult and imprecise [118]. Wearing of face masks will reduce the risk. During the COVID-19 pandemic, the airline industry has taken a pro-active approach to increase passenger safety through extended ventilation at the gate, boarding and deplaning strategies, improved aircraft disinfection, and pre-flight screening such as temperature checks and COVID-19 testing [119]. Of the other respiratory infections, the common cold is too common for in-flight transmission to be studied, and measles and meningococcal infection are known to be transmitted occasionally.

Food- and water-borne transmission usually involves a single source that transmits microbes to many people. The most commonly reported diseases transmitted on aircraft have been due to contaminated food. Vector-borne transmission is actually via insects, though theoretically might be via vermin (as with plague rats on ships), and aircraft disinsection is the way to address this problem [117].

## Conclusions

COVID-19 is the worst pandemic in scale and speed of this century associated with the highest number of global deaths, with most of the deaths reported in high income countries. Risk factors such as increasing age, obesity, and comorbidities including pulmonary diseases, diabetes, cancer and neurological diseases drive the infection fatality rate. Although the infection fatality rate is far lower compared to other emerging infectious diseases such as Ebola or yellow fever, the global toll in terms of deaths is far higher due to its propensity of high secondary attack rates with a high basic reproduction number. Its rapid spread via air travel around the world was relentless despite early travel restrictions and travel bans. However, travel restrictions delayed the importation and reduced the outbreak size. Mobility restrictions continue to be used across countries. Due to the high reproduction number, combating COVID-19 will require an all-society and all-government approach.

In contrast, travel restrictions for arboviral diseases will be ineffective as the focus is on vector control. The re-emergence of measles requires addressing declining vaccine coverage rates, and will not require travel restrictions. Emerging infectious such as Ebola, monkeypox, and poliomyelitis will not benefit from travel restrictions as the risk of spread via travel is minimal, and effective tools in terms of vaccines are available. H1N1 as a respiratory pathogen was spread rapidly around the globe, even faster than COVID-19, but is associated with a much lower infection fatality rate, and was less driven by clustering effects and mass gatherings than COVID-19.

## Data Availability

All available
